# *In vivo* evaluation of porous nanohydroxyapatite/polyamide 66 struts in a goat cervical fusion model

**DOI:** 10.1038/s41598-020-65341-1

**Published:** 2020-06-26

**Authors:** Xi Liang, Feilong Li, Xuan Gong, Junchao Li, Shijie Yin, Qi Li, Ziming Liu, Zenghui Zhao, Xiaolin Tu, Wei Huang, Ning Hu

**Affiliations:** 1 Department of Orthopedics, The First Affiliated Hospital of Chongqing Medical University, Chongqing, 400016 China; 2 Department of Orthopedics, The People’s Hosptial of Dazu District, Chongqing, 402360 China; 3 Department of Nursing, Chongqing General Hospital, Chongqing, 400013 China; 4 College of Material Science and Engineering, Chongqing University, Chongqing, 400044 China; 5 Department of Orthopedics, The University of Hong Kong-Shenzhen Hospital, Shenzhen, 518053 China; 6 Department of Orthopedics, Chongqing Beibei Traditional Chinese Medical Hospital, Chongqing, 400700 China; 7 Laboratory of Skeletal Development and Regeneration, Institute of Life Sciences, Chongqing Medical University, Chongqing, 400016 China

**Keywords:** Electron microscopy, Bioinspired materials

## Abstract

The hollow cylindrical nanohydroxyapatite/polyamide 66 strut (n-HA/PA66) has been used clinically for anterior cervical reconstruction. However, rates of occurrence of a “radiolucent gap” between the dense strut and adjacent endplates were reported. The aim of this *in vivo* study was to evaluate the viability and advantages of the novel porous n-HA/PA66 strut. The goat C3/4 partial discectomy and fusion model was built, and two groups of n-HA/PA66 struts were implanted into C3/4: group 1, porous n-HA/PA66 strut; and group 2, hollow cylindrical n-HA/PA66 strut filled with autogenous cancellous bone. CT evaluation was performed to assess the fusion status after 12 and 24 weeks. The cervical spines were harvested. Histomorphological analysis was performed to determine new bone formation. Biomechanical testing was performed to determine range of motion (ROM). CT confirmed the disappearance of the boundary of the porous strut and host bone, while the radiolucent gap remained clearly discernible in the dense strut group. The mean CT fusion scores of the porous group were significantly higher. Histologic evaluation showed that the porous struts promoted better osteointegration. Calcein fluorochrome labelling indicated faster bone ingrowth in the porous struts. Biomechanical tests revealed that the porous struts had significantly reduced micromotion. The porous n-HA/PA66 strut could offer interesting potential for cervical reconstruction after corpectomy.

## Introduction

Anterior cervical discectomy and fusion (ACDF) has been highly successful in treating cervical discogenic diseases associated with radiculopathy or myepathy^1^. Many struts have been used to reconstruct the stability of the anterior cervical column, including structural autografts, allografts, struts and synthetic substitutes^2,3^. However, the optimal surgical procedure remains controversial^4,5^. Autogenous tricortical iliac crest grafts have been reported to cause donor site morbidity in approximately 25% of cases, such as infection, persistent donor site pain and hematoma^6^. Lower fusion rates, higher rates of breakage, poor biocompatibility, immunologic rejection and the risk of disease transmission greatly limited the use of structural allografts^7,8^. The complications of subsidence, stress shielding, and radiopacity of titanium mesh struts are still of great concern, hindering them from becoming ideal reconstructing devices^9^.

Inorganic HA and organic polymers constituted by various composite materials have been explored as bone substitutes over the years^2^. Anterior cervical reconstruction was performed using a hollow cylindrical nanohydroxyapatite/polyamide 66 strut (n-HA/PA66, Sichuan National Nano Technology Co., Ltd. Chengdu, Sichuan) for many years. The n-HA/PA66 strut is a non-metallic strut device; it is a composite material of nanohydroxyapatite and polyamide 66, and the device is similar to an apatite blend with collagen organisms in natural bone^10^. Some studies have reported satisfactory clinical outcomes^1,2,5,11^. However, a previous clinical study recorded the occurrence rate of the “radiolucent gap” between this dense n-HA/PA66 strut with a hollow design and the adjacent endplate at the one-year and last follow-up, with rates of 56% (28/50) and 62% (31/50), respectively^2^. The subsidence rate was 4% (2/50) at the one-year follow-up, and at the final follow-up, the rate was increased to 8% (4/50). The possible reason may be that the osteoconductivity was insufficient in the n-HA/PA66 composite and the radiographic penetrability of the fibrous tissue exhibited a “radiolucent gap”^2^.

In order to solve these shortcomings, a novel porous n-HA/PA66 composite has been developed in recent years^12–14^. Structural 3D bioactive scaffolds with suitable interpenetrating porosity and mechanical stability meet several requirements considered essential for bone regeneration. High porosity and proper pore size may facilitate osteocyte seeding, differentiation, growth, survival and proliferation^15^. The objective of this study was 2-fold: first, we evaluated the biocompatibility, osteogenesis and biomechanical properties of the porous n-HA/PA66 strut *in vivo* using a goat cervical discectomy model. The second objective was to explore comparative assessments of the porous n-HA/PA66 strut with the dense hollow cylindrical n-HA/PA66 strut.

## Methods and Materials

### Ethics statement

The use of animals and the experimental protocols were approved by the Institutional Review Board and the Animal Care Committee of Chongqing Medical University (Approval No. 201213). All methods were carried out in accordance with the approval ethical guidelines.

### Materials

**Figure 1:**
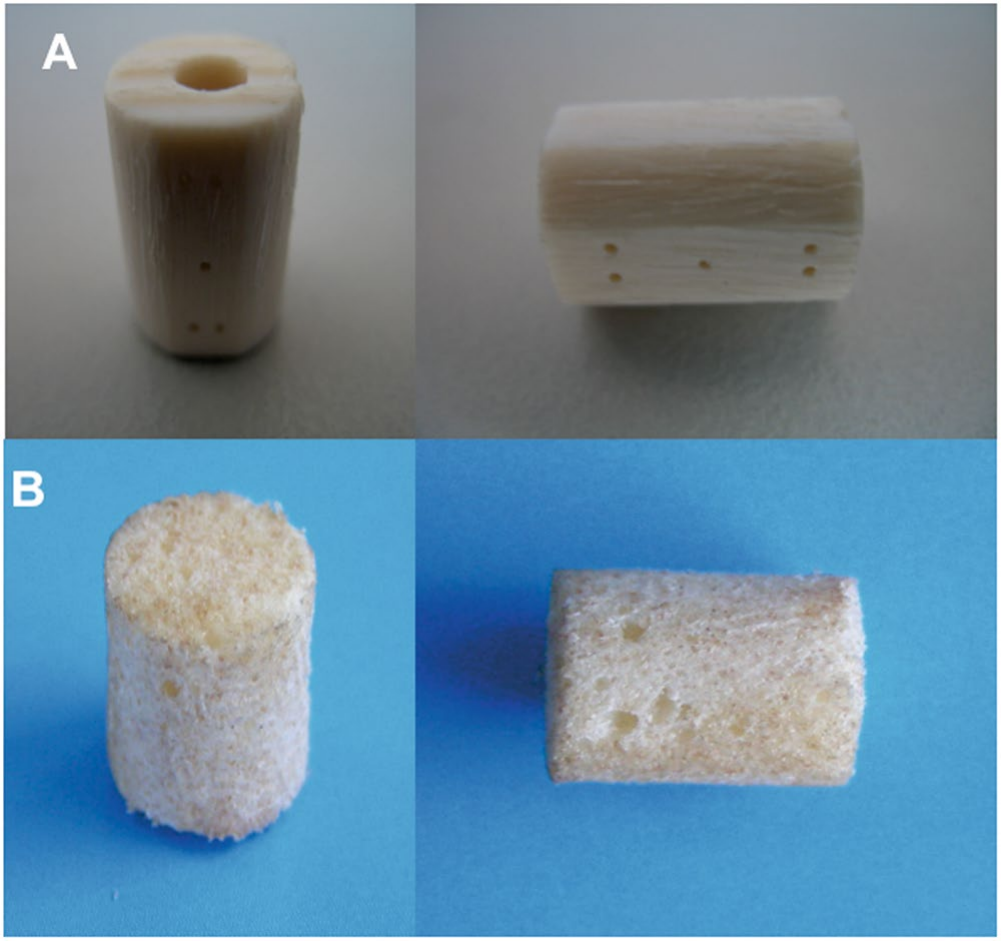
Implants tested in the study. (**A**) The dense nanohydroxyapatite/polyamide 66 struts (n-HA/PA66) is a hollow cylinder 9 mm in outer diameter and 3 mm in inner diameter, with several 0.5-mm holes and slots around the outside. (**B**) The porous n-HA/PA66 strut is 9 mm in diameter. The porosity, average pore diameter and HA content were 80%, 300 μm and 40 wt%, respectively. Both were designed and fabricated jointly by the Institute of Materials Science and Technology of Sichuan University and our department.

The dense n-HA/PA66 strut with hollow cylindrical design and the porous n-HA/PA66 composite strut were designed and fabricated in collaboration with the Chinese Sichuan University Institute of Materials Science and Technology department and our department. The hollow n-HA/PA66 strut has been approved for clinical use since 2005 by the State Drug and Food Administration of China^1,2,5,11^. The n-HA/PA composites were developed with a bioactive apatite (n-HA) and organic polymer (PA) to mimic natural bone^12^. The hollow cylindrical strut was designed with a 9-mm outer diameter, a 3-mm inner diameter and a 10-mm length, with several 0.5-mm holes and slots around the outside (Fig. 1A). Each strut had grooves at each end to increase the friction between the strut and vertebra.

The dimensions of the porous n-HA/PA66 struts used in the experiment were 9 mm in diameter and 10 mm in length (Fig. 1B). The porosity of the struts was 80%, the average pore diameter was 300 μm, and the HA content was 40 wt%. The previous results of mechanical testing showed that the strengths of bending, tensing, and compressing were 68, 50 and 95 MPa, respectively, and those strengths were matched with human natural cortical bone.

### Animals

Thirty skeletally mature goats (females, age 2-3 years, body weight 20-25 kg, provided by the Experimental Animal Center of Chongqing Medical University) were divided into two groups: one group for the implantation of dense n-HA/PA66 struts with hollow cylindrical design and another for the novel porous n-HA/PA66 composite struts.

### Surgical technique and group management

**Figure 2:**
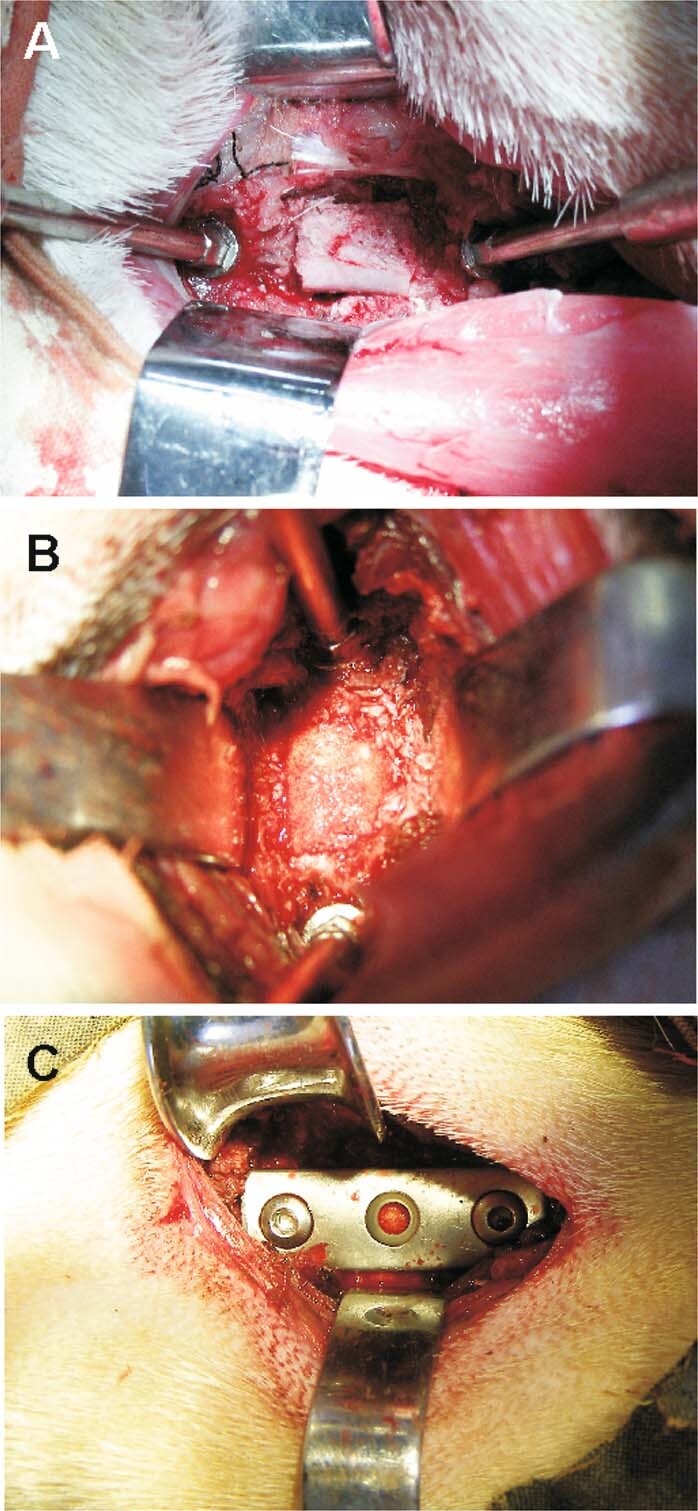
Different implants in the surgery. (**A**) A dense n-HA/PA66 strut was filled with autogenous cancellous bone. (**B**) A porous n-HA/PA66 strut. (**C**) Additional stabilization with a 3-hole plate.

All surgeries were performed by one senior spine surgeon. A right-sided anterior cervical approach was used. Ketamine (10 mg/kg) and diazepam (0.15 mg/kg) were used to induce anaesthesia through intravenous administration, maintained with endotracheal inhalation of 1.5% isoflurane throughout the operation. All animals were placed in the supine position with neck extension. Following the discectomy of the cervical disc between C3 and C4, discectomy was performed in line with the technique of Smith and Robinson^16,17^. The superior and inferior endplates were elaborately made by high-speed deburring and scraping to remove covered cartilage. The dense n-HA/PA66 strut was filled with autogenous cancellous bone from the resected vertebra, and the strut was implanted into the intervertebral space after corpectomy (Fig. 2A). The appropriately sized porous n-HA/PA66 strut was inserted into the prepared intervertebral space (Fig. 2B). To achieve immediate stabilization, a 3-hole compression plate was used to fix the C3 and C4 vertebral bodies (Fig. 2C). To reduce the perioperative infection risk, the goats received antibiotic prophylaxis (penicillin solution, 800,000 units per day, muscular injection). After surgery, all animals were required to wear a soft cervical collar for approximately four weeks. The diet, activity level, wound healing, and range of motion were assessed for each goat.

### Radiographic analysis

**Figure 3:**
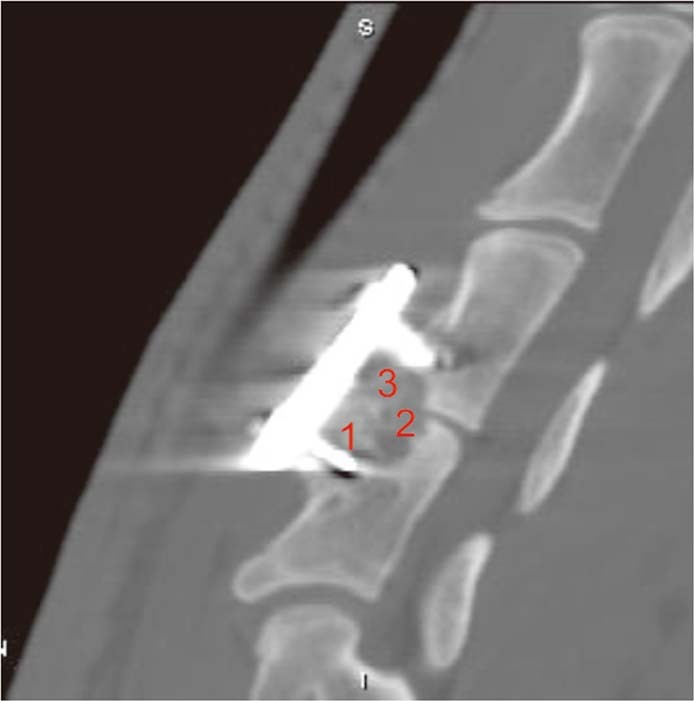
Radiographic assessment. Four sagittal planes at the C3 and C4 segments were reconstructed with 2-mm slice thickness. The interface between the implant and vertical bone on each plane was divided into 1, 2 and 3 zones for the degree of fusion status. The scoring criteria were described by van Dijk and graded as follows: 0= pseudoarthrosis; 1= ingrowth of bone with the strut securely fixed to vertebral bone above and below, but with a radiolucent discontinuity in the fusion mass; 2= arthrodesis with solid bone bridging the fusion area. The total points for each sample was 24 points.

Radiographic assessment was completed by two independent observers. We evaluated the presence of a “radiolucent gap” between the n-HA/PA66 struts and their contacted endplates to assess the osteoconductivity and osseointegration. Each group of fifteen samples at 12 weeks and each group of ten samples at 24 weeks underwent computed tomography (CT) scans of C3 and C4 using a 64-detector row CT system (Somatom Sensation 64, Siemens Medical Imaging, Erlangen, Germany) postoperatively. Four sagittal planes were reconstructed with 2-mm slice thickness. The interface between the implant and vertical bone on each plane was divided into 1, 2 and 3 zones for the degree of fusion status (Fig. 3). Fusion was defined by continuous bridging of trabecular bone and the absence of radiolucent lines on CT^18,19^. All the radiographs were encoded and reviewed in a blinded fashion to assess interbody fusion according to a three-point radiographic score (RS) that was described by van Dijk *et al.*^5,20,21^. RS0 indicates pseudoarthrosis; RS1 indicates ingrowth of bone with the strut securely fixed to vertebral bone above and below but with a radiolucent discontinuity in the fusion mass; and RS2 indicates arthrodesis with solid bone bridging the fusion area^5^. The total points for a section was 24 points^5^.

### Gross and histological observations

Five goats randomly selected from each group were humanely euthanized with diazepam and KCI overdose at 12 and 24 weeks after implantation. Their cervical columns were harvested, including segment C3-C4 with all ligamentous structures. General photography was conducted to observe the interface between implants and vertebral bone. The C3/C4 motion segments were fixed in 10% normal buffered formalin for 2–4 weeks, dehydrated in ascending concentrations of ethanol and cleared in xylene for 4 days, followed by embedding undecalcified in isobornyl methacrylate (Technovit 1200 VLC, Kulzer, Algol, Germany). Specimens were cut into approximately 5-μm-thick sections with a Leica heavy duty sliding microtome (Leica, Wetzlar, Germany), and the sections were stained with haematoxylin and eosin (H&E) and Masson’s trichrome stains^22,23^. The Olympus B51 virtual microscope with a U-CMAD3 camera attached (Olympus Optical Co. Ltd, Tokyo, Japan) were used to image the histologic sections.

### Quantification of newly formed bone

We statistically analysed the histological sections of different implantation periods to quantitatively determine the amount of newly formed bone from the randomly euthanized five goats of each group. Three pieces were randomly chosen from both the hollow cylindrical strut group and porous strut group from every implantation period histological section (12 and 24 weeks). After H&E staining, each section was observed under a light microscope at 100 x magnification, and at least 10 images were randomly obtained from one section. New bone volume (NBV) was expressed as the percentage of newly formed bone area in the implants and analysed by Image-Pro Plus (Media Cybernetics, USA) software.

### Calcein labelling observation

Before histological evaluation of bone formation rates on the 7th day, fluorochrome labels (calcein), when bound to calcium ions, can be incorporated at sites of the mineralization front in the form of hydroxyapatite crystals. Calcein injections (C-0875; Sigma-Aldrich Chemie, Germany) were given twice, subcutaneously at 20 mg/kg^23^. Hard tissue sections were left unstained to study calcein labelling under UV light.

### Biomechanical test

**Figure 4:**
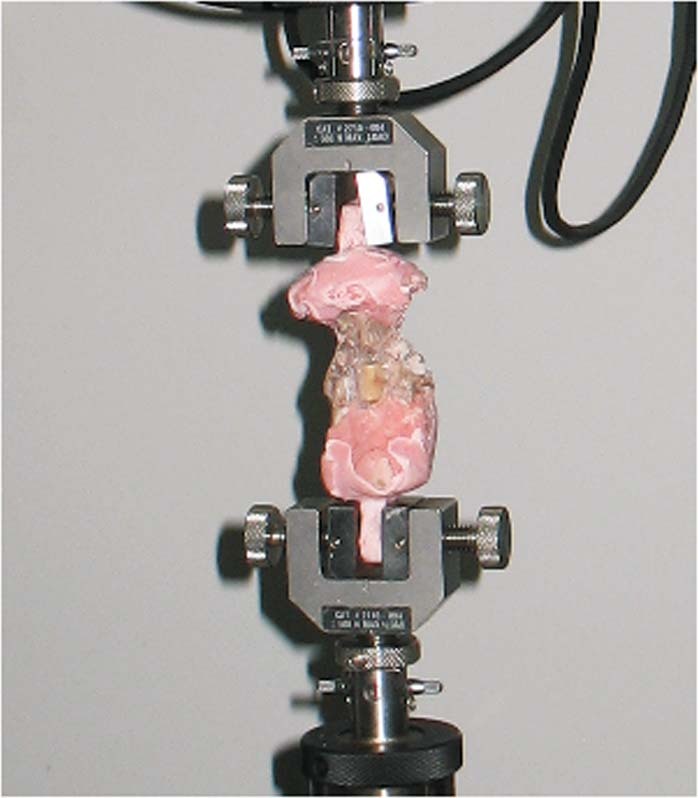
Biomechanical experimental setup for applying pure moments to the cervical segment. The sample was placed on a support jig mounted on the universal mechanical testing machine.

Five goats selected from each group were sacrificed at 24 weeks after surgery. The C3/C4 motion segment was isolated from the cervical spine and cleaned of all residual musculature, metallic plates and screws, with care taken to preserve all ligamentous structures. Biomechanical testing was completed within 24 hours. We performed the test as described by Zhou^24^. A mechanical testing system (MTS) machine (Instron, Model 8874, USA) was used to perform nondestructive biomechanical testing. The vertebrae were mounted in pots using polymethylmethacrylate. The lower pot was rigidly attached to the base of the testing apparatus (Fig. 4). The weight of the upper fixation pot represented the average weight of the goat head, resulting in a compressive preload of 25 N. Pure bending moments induced flexion, extension, left and right lateral bending, and left and right axial rotation. Moments were applied in a quasistatic manner in increments of 1 Nm to a maximum of 6 Nm. Specimens were preconditioned with three cycles of 6-Nm load with a velocity of 1.2 mm/s of the mobile bar. We measured the fourth cycle. The motional analysis system tracked the resultant three-dimensional range of motion (ROM) of each segment.

### Statistical analysis

We performed all quantitative experiments three times. The data are expressed as the mean ± s.d., and statistical significance between two groups was determined by one-way analysis of variance and Student’s t-test. The level of significance was determined at P < 0.05.

## Results

### Macroscopic observation

**Figure 5:**
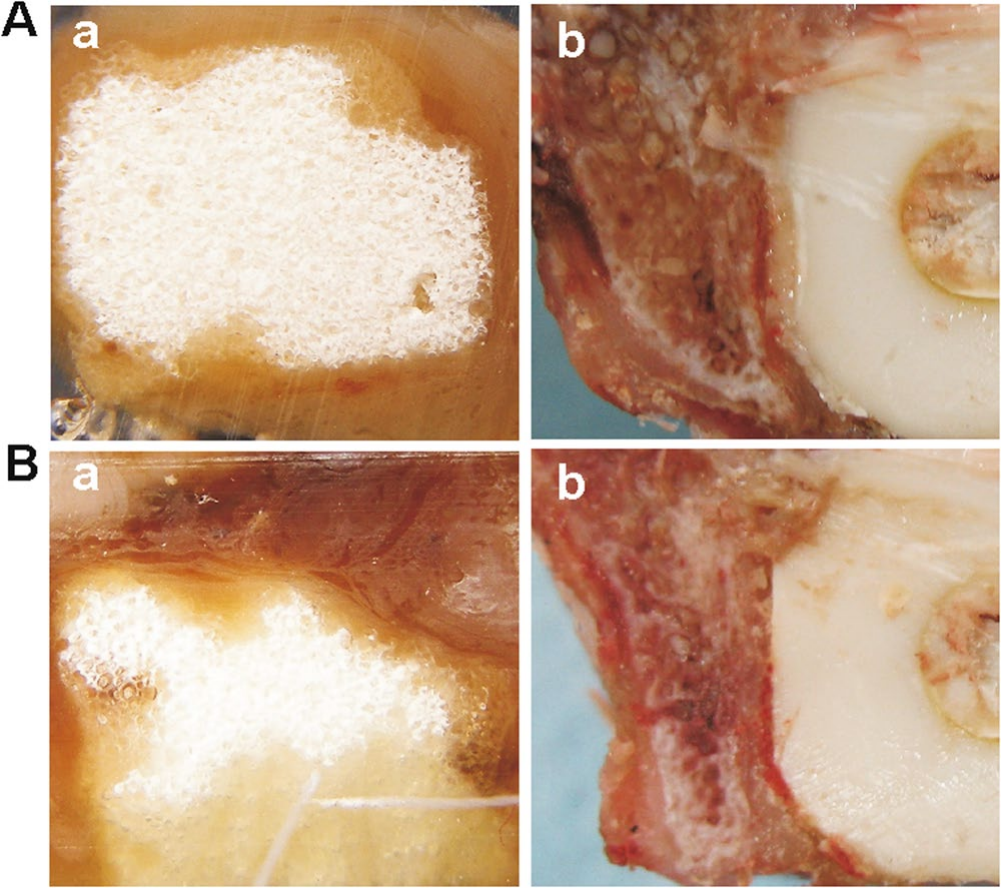
Representative macroscopic photographs. (Aa) The porous strut group at 12 weeks post-implantation; (Ab) The dense strut group at 12 weeks post-implantation; (Ba) The porous strut group at 24 weeks; (Bb) The dense strut group at 24 weeks.

No goats died in any of the groups. Food intake in each goat gradually improved. All wounds healed to grade A. No postoperative infections, allergic reaction, or material displacement were encountered. At 12 weeks post-implantation, the strut and the vertebral body were closely integrated in both groups. In the dense strut group, fibrous callus formation was observed at the interface, and some gaps between the strut and the host bone were still visible. In the porous strut group, the boundary between the strut and host bone became unclear, the sufficient formation of mature bone tissues was observed, and the bone tissues were ingrown into the pores of the artificial scaffold and bonded tightly with the material (Fig. 5A a and b). At 24 weeks, the interface between the material and host bone formed a close union in both groups. In the dense strut group, more mature callus was detectable. In the porous strut group, new bone regenerated and penetrated through the interconnective pores to the scaffolds (Fig. 5B a and b).

### Radiographic analysis

**Figure 6:**
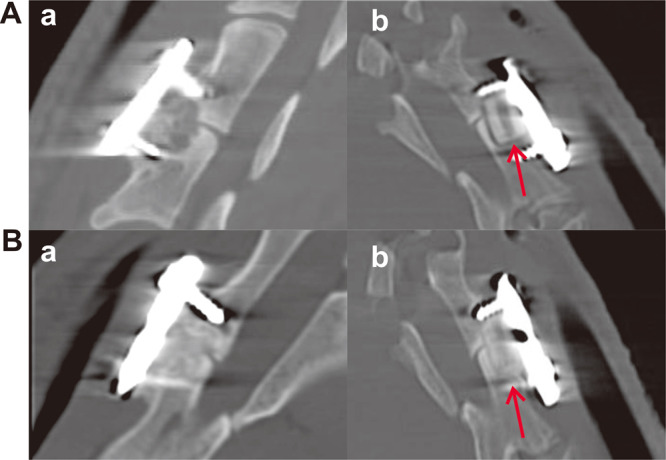
Lateral view scans of the goat C3/C4 motion segment in each group with the different implants. (Aa) The porous strut group at 12 weeks post-implantation; (Ab) The dense strut group at 12 weeks post-implantation; (Ba) The porous strut group at 24 weeks; (Bb) The dense strut group at 24 weeks. A radiolucent gap was observed at the conjunction site (red arrows).

The evaluation of radiographic outcomes included the radiolucent gap and fusion rate. In both groups, the interface between the implants and the host bone was clearly discernible at 12 weeks after the operation (Fig. 6A a and b). At 24 weeks after implantation of the porous n-HA/PA66 strut, the disappearance of the boundary of the porous strut and host bone indicated that new bone had grown into the strut, and the density of newly formed bone was similar to that of host bone. Almost complete osteointegration of the material/bone boundary had been achieved. In the dense control group, the longitudinal hole in the centre of the strut became unidentifiable, which illustrated that more bone tissue grew. However, the radiolucent gap remained clearly discernible (Fig. 6B a and b). CT microradiographic analysis also confirmed the histological study results.

**Figure 7:**
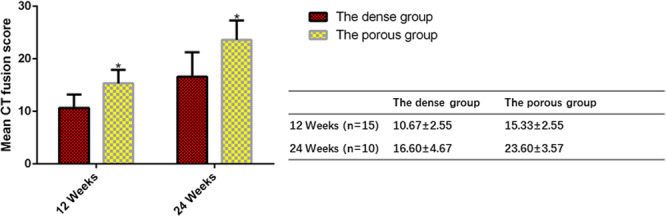
The mean CT fusion scores of the porous strut were significantly higher than those of the dense strut group. The mean CT fusion scores of each group at 12 and 24 weeks after surgery are expressed as the mean ± s.d and histogram, bars represent standard deviation values. *p < 0.05.

The mean CT fusion scores of each group at 12 and 24 weeks after surgery are shown (Fig. 7). Noticeable differences in the fusion rate were found between the two groups. In comparison with the results of the dense group, those of the porous group were superior (Fig. 7).

### Histological observation

**Figure 8:**
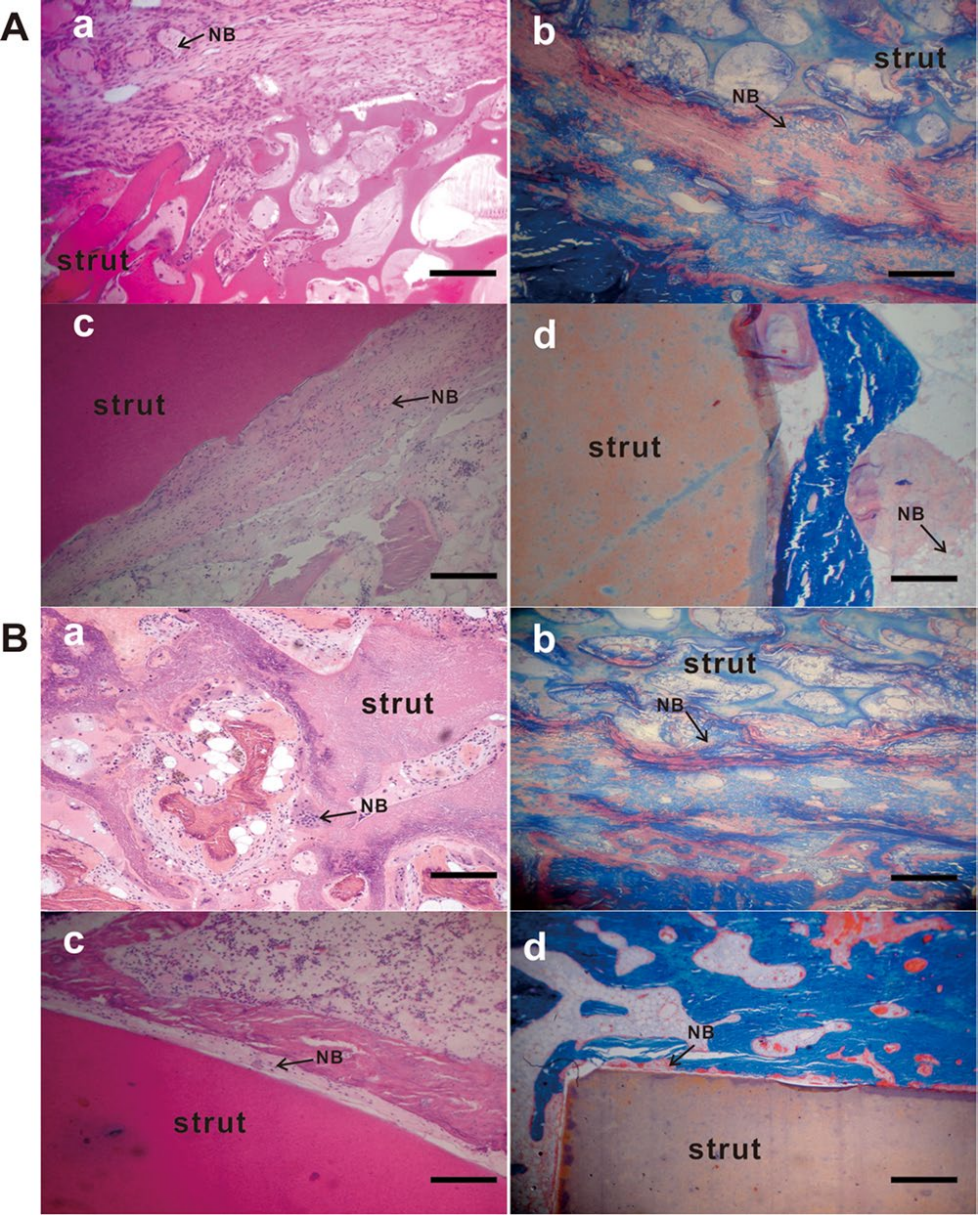
Histologic analysis of the retrieved samples. The retrieved samples were fixed, decalcified, paraffin-embedded and subjected to H&E staining (Aa and c, Ba and c) and Masson’s trichrome staining (Ab and d, Bb and d). Magnification, 100×. (Aa and b) The porous n-HA/PA66 strut group at 12 weeks after surgery; (Ac and d) The dense strut group at 12 weeks after surgery. (Ba and b) The porous strut group at 24 weeks; (Bc and d) The dense strut group at 24 weeks. Scale bar = 100 µm. NB, new bone.

All the struts implanted after 12 weeks were encapsulated by fibrous callus. A large quantity of fibrous tissue and osteoblasts were observed along the interface, proving the formation of new bone. Moreover, in the porous n-HA/PA66 strut group, a large proportion of osteoblasts penetrated the interconnective pores, which may accelerate the mineralization and regeneration of bone. The interface between the porous material and the host bone was hardly detectable and formed a close union without any gap (Fig. 8A a and b). In the dense n-HA/PA66 strut group, the neighbouring bone tissue surrounding the strut seemed conspicuous. Few qualities of new bone had ingrown into the strut (Fig. 8A c and d).

At 24 weeks after surgery, more mature trabecular tissues were deposited at the interface of the struts and host bone, and active osteoblasts were present in both groups (Fig. 8B). In the porous group, the sufficient formation of mature osseous tissue crept into the interconnected porosity and connected tightly with the material directly (Fig. 8B a and b).

### Quantification of newly formed bone

**Figure 9:**
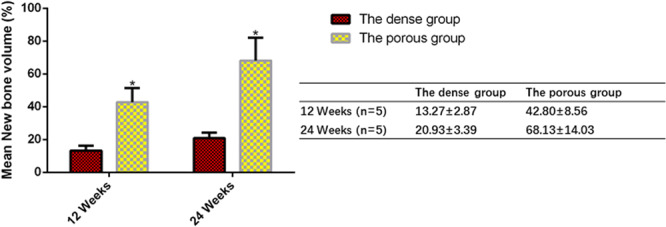
Quantification of newly formed bone within the implants at various implantation periods. The mean NBV data of each group at 12 and 24 weeks after surgery are expressed as the mean ± s.d and histogram, bars represent standard deviation values. *p < 0.05.

We performed a quantitative determination of newly formed bone through statistical analysis of histological sections. Figure 9 shows the new bone volume (NBV) at each implantation period. Obviously, at 12 and 24 weeks post-implantation, the amounts of newly formed bone in the porous n-HA/PA66 strut group increased dramatically, much more than that of the dense n-HA/PA66 strut with hollow cylindrical design. The NBV in the inside pores was greater in the porous n-HA/PA66 strutsthan in the dense n-HA/PA66 struts. New bone formation gradually grew in the porous n-HA/PA66 struts. Histological assessment revealed that bone formation was more active in the porous group than in the dense group.

### Calcein staining of new bone formation

**Figure 10:**
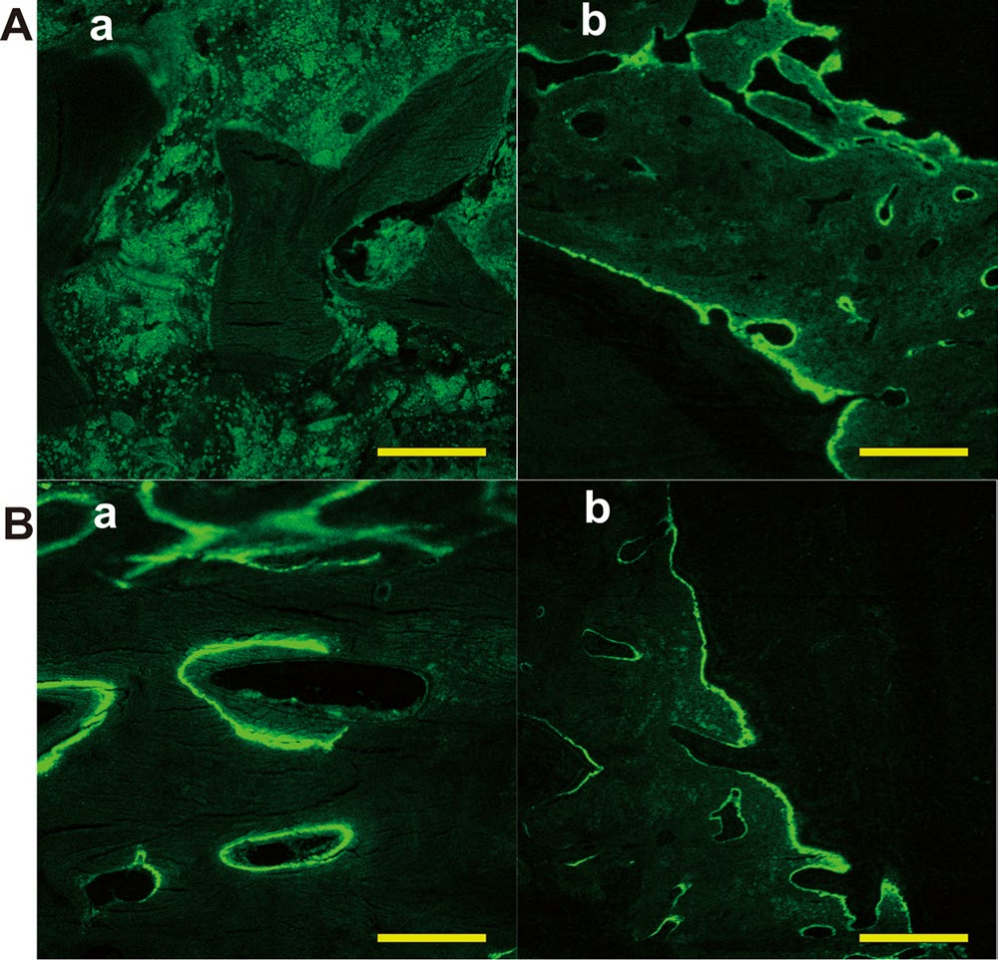
Representative calcein-labelled images show greater mineralized bone (green) deposition for the porous strut group compared to the dense strut group implants. Magnification, 400×. (Aa) The porous n-HA/PA66 strut group at 12 weeks after surgery; (Ab) The dense strut group at 12 weeks after surgery. (Ba) The porous strut group at 24 weeks; (Bb) The dense strut group at 24 weeks. Scale bar = 100 µm.

To further discover the difference in new bone development on the struts, we applied fluorescent calcein green to label the newly formed bone. Both groups exhibited varying amounts of woven bone formation characterized by continuous fluorescent lines at 12 weeks (Fig. 10A a and b). In the 24-week specimens, the diffuse fluorescence areas of calcein green partially decreased (Fig. 10B a and b). Calcein-labelled bones showed that progressive bone remodelling continued at 3 months. A substantial slowing from the 6-month time point was observed from the number of active sites noted on bone sections. For the porous n-HA/PA66 strut group, the fluorochrome bands were wider and loosely mixed. Furthermore, the labelling was not limited to the outer regions of the porous struts adjacent to the strut-bone interface but also appeared multifocal at different pore areas of the implants. However, the fluorescent bands were weakly and sparsely marked and limited to the outer regions of the dense strut group. Calcein fluorochrome labelling (green) of all unstained histologic sections illustrated the dynamic deposition of mineralized bone matrix at the interface and internal pore structure of the porous HA/PA66 strut.

### Biomechanical evaluation

**Figure 11:**
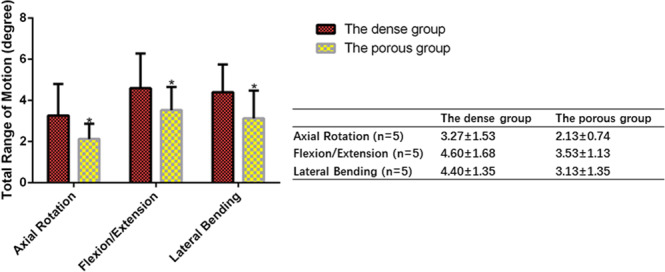
Range of motion (ROM) measurements at 24 weeks using three different test modes (left/right axial rotation, flexion/extension, and left/right lateral bending). In all test modes, ROM values were significantly lower in the porous strut group than those in the dense strut group, showing that porous struts achieved better fusion quality with higher mechanical stability. The mean ROM measurements of each group are expressed as the mean ± s.d and histogram, bars represent standard deviation values *p < 0.05.

The mechanical stability of cervical fusion was evaluated by measuring the relative micromotion of two fused vertebral bodies through the ROM assay. The extent of this motion decrease was associated with the fusion stability. After 24 weeks, the implantation of the porous HA/PA66 strut showed a significantly lower ROM than the dense n-HA/PA66 strut with hollow cylindrical design for all the axial rotation, flexion-extension, and lateral bending testing modes (P < 0.05), demonstrating that the porous HA/PA66 strut produced a much better fusion quality and a higher mechanical stability (Fig. 11).

## Discussion

Cervical spine reconstruction is much more difficult after corpectomy than discectomy. Many surgeons prefer the struts, titanium mesh cages (TMCs), packed with cancellous bone for cervical fusion. TMCs can provide effective support and satisfactory fusion without using structural iliac autografts. Donor-site morbidity and the risk of disease transmission associated with allografts have been prevented^7,10,25^. However, the typical complication, TMC subsidence, has been frequently observed in the early postoperative period^1,8–10,26,27^. Chen *et al*. reported that TMC subsidence (more than one millimetre) occurred in 79.7% of patients, and 19% of cases who underwent TMC reconstruction after anterior cervical corpectomy had severe subsidence (more than three millimetres)^9^. Weber *et al*. found that 18 patients who underwent primary cervical corpectomy had an average graft subsidence of 2.9 mm, and 11.1% needed a revision program^28^. It seems that TMCs lead to height loss and segmental alignment, and they also increase the possibility of fusion failure and serious neurological complications. However, a systematic review of the incidence and clinical relevance of TMC subsidence in anterior cervical discectomy and fusion reported that the mean incidence of subsidence was 21% using strut screw combinations, PEEK, titanium, or PMMA struts^29^. The subsidence rate of titanium struts was 24.9%, and the risk for subsidence using titanium struts also seems lower^29^. Titanium mesh struts subsidence-related questions remain controversial, but subsidence has resulted in serious consequences, such as buckling of the cervical ligamentum flavum, foraminal stenosis and re-compression of the cervical spinal cord and nerve roots^26,30–32^.

Anterior reconstruction biomaterials have been developed for better clinical outcomes^33^. The aim of artificial struts is not only to provide sufficient mechanical strength, great histocompatibility and appropriate elasticity but also to integrate with host bone. Requisite durability and functionality are governed by the properties of biomaterials. The composite, n-HA/PA66, is made by infiltrating nano-HA into PA66. Because it mimics natural bone in that apatite is distributed within a collagen matrix^5,34,35^, the n-HA/PA66 composite has the mechanical strength of HA and the elastic properties of PA66^5,13,33^. The dense hollow cylindrical n-HA/PA66 strut was designed for use in 2005, and clinical follow-up has gradually been reported in recent years^1,2,5,11^. Zhao *et al*. reported a fusion rate of 94.3% in their 35 patients with a hollow cylindrical n-HA/PA66 strut fusion following cervical corpectomy^1^. Zhang *et al*. found that using a n-HA/PA66 strut could result in a lower subsidence incidence, better fused segment height maintenance and similar bony fusion as TMCs in 117 patients with multilevel cervical spondylotic myelopathy who underwent anterior cervical corpectomy and fusion^5^. However, Zhang *et al*. noted that the incidences of a “radiolucent gap” at the interface between this dense n-HA/PA66 strut with a hollow design and the vertebra were 56% (28/50) at one year and 62% (31/50) at the last follow-up^2^. He thought the radiographic penetrability of the fibrous tissue exhibited a “radiolucent gap”^2^. Xiong *et al*. indicated that it had a high loss rate if the fibrous tissue growth wrapped the interface of the bone implant, which could lead to failure^12^.

Bone tissue engineering using porous biomimetic matrices coupled with various modifications has been a concern in many studies^36^. A porous bone substitute provides a three-dimensional interconnected pore structure for cell adhesion, migration and proliferation of new bone tissue^37^. Our result was in accordance with the finding of Li *et al*, who showed that the cylindrical n-HA/PA66 scaffold, designed as parallel linear pores with a diameter of 300 μm, was the best choice for bone regeneration and infiltration^38^. Jie and Li mentioned that the more than 50% porosity of the porous n-HA/PA66 strut had valuable effects on osteocyte adherence and extracellular matrix deposition on its inner surface^39^. Li *et al*. stated that the inner porous surface enables cells such as osteoblasts to colonize it^40^. It also acts as an organizer of vascular canals. The interconnective porous structure increased the transmission of oxygen and nutrients and induced the growth of blood vessels and nerves^12,40^. Xiong and Wang mentioned that a three-dimensional structure with interconnective pores could promote cell adhesion, differentiation, and proliferation; it also promoted fibrovascular and nerve colonization, which caused the new bone “crawls” into the material to achieve early osteoinduction^12,13^. The outer layer, which is dense or less porous, can prevent osteoblast ingrowth and mesenchymal stem cell growth into biomaterials. It was suggested that the porous n-HA/PA66 strut with high interpenetrating porosity appeared to promote rapid tissue infiltration and bone tissue regeneration, whereas this process was delayed in the dense strut. Zhong *et al*. found a common n-HA/PA66 strut subsidence in patients undergoing anterior cervical corpectomy decompression and fusion. They found that postoperative fused segments have height reduction and that height reduction is an independent risk factor for n-HA/PA66 strut subsidence^41^.

The pore structure theoretically reduces the rigidity of the biomaterials that are close to the elastic modulus of the bone tissue, which ensures that the interface is stable^15^. The designed increase in pore size, but not the porosity, decreases the mechanical strength. The average pore size of the porous n-HA/PA66 strut material used in the study was 300 µm, and the porosity was 80%. Our mechanical results of the porous n-HA/PA strut showed that the composite has a compressive strength of 95 MPa, which is similar to the lower value of the cortical bone. The porosity rate was 80%, which was an appropriate porosity that satisfied the needs of cell infiltration and proliferation and ingrowth. We found that when the porous n-HA/PA66 struts were implanted, the pores of the struts and host bone gradually integrated into the cross-linked form. The porous HA/PA66 strut presented better biocompatibility and stronger osteogenesis of bone formation than the dense strut. The highly interconnected pore structure may be beneficial for forming mechanical locking and anchoring it with the surrounding bone^37^. However, Zhong *et al*. suggested that the n-HA/PA66 strut should be further optimized and that more detailed measurements of the distraction should be performed before clinical applications^41^. Much scrutiny needs to be executed in future work to avoid poor clinical outcomes.

## Conclusion

Compared with the dense n-HA/PA66 strut with a hollow cylindrical design, the porous n-HA/PA66 strut effectively improved intervertebral bony fusion and offers interesting potential for cervical reconstruction after corpectomy.
